# Alleviative effects of pinostrobin against cadmium-induced renal toxicity in rats by reducing oxidative stress, apoptosis, inflammation, and mitochondrial dysfunction

**DOI:** 10.3389/fnut.2023.1175008

**Published:** 2023-05-24

**Authors:** Muhammad Umar Ijaz, Sabahat Shahzadi, Ali Hamza, Rabia Azmat, Haseeb Anwar, Tayyaba Afsar, Huma Shafique, Mashooq Ahmad Bhat, Ahmed M. Naglah, Mohamed A. Al-Omar, Suhail Razak

**Affiliations:** ^1^Department of Zoology, Wildlife and Fisheries, University of Agriculture, Faisalabad, Pakistan; ^2^Department of Physiology, Government College University, Faisalabad, Pakistan; ^3^Department of Community Health Sciences, College of Applied Medical Sciences, King Saud University, Riyadh, Saudi Arabia; ^4^Institute of Cellular Medicine, Newcastle University Medical School, Newcastle University, Newcastle upon Tyne, United Kingdom; ^5^Department of Pharmaceutical Chemistry, College of Pharmacy, King Saud University, Riyadh, Saudi Arabia

**Keywords:** cadmium, pinostrobin, mitochondria, renal damage, apoptosis

## Abstract

**Introduction:**

Cadmium (Cd) is a highly toxic heavy metal that can be found everywhere in the environment and can have harmful effects on both human and animal health. Pinostrobin (PSB) is a bioactive natural flavonoid isolated from *Boesenbergia rotunda* with several pharmacological properties, such as antiinflammatory, anticancer, antioxidant, and antiviral. This investigation was intended to assess the therapeutic potential of PSB against Cd-induced kidney damage in rats.

**Methods:**

In total, 48 Sprague Dawley rats were divided into four groups: a control, a Cd (5 mg/kg), a Cd + PSB group (5 mg/kg Cd and 10 mg/kg PSB), and a PSB group (10 mg/kg) that received supplementation for 30 days.

**Results:**

Exposure to Cd led to a decrease in the activities of catalase (CAT), glutathione reductase (GSR), superoxide dismutase (SOD), and glutathione peroxidase (GSH-PX), whereas levels of reactive oxygen species (ROS) and malondialdehyde (MDA) increased. Cd exposure also caused a substantial increase in urea, kidney injury molecule-1 (KIM-1), neutrophil gelatinase-associated lipocalin (NGAL), and creatinine levels. Moreover, a noticeable decline was noticed in creatinine clearance. Moreover, Cd exposure considerably increased the levels of inflammatory indices, including interleukin-1b (IL-1b), tumor necrosis factor-a (TNF-a), interleukin-6 (IL-6), nuclear factor kappa-B (NF-kB), inducible nitric oxide synthase (iNOS), and cyclooxygenase-2 (COX-2) activity. Cd treatment decreased the expression of the antiapoptotic markers (Bcl-2) while increasing the expression of apoptotic markers (Bax and Caspase-3). Furthermore, Cd treatment substantially reduced the TCA cycle enzyme activity, such as alpha-ketoglutarate dehydrogenase, succinate dehydrogenase, malate dehydrogenase, and isocitrate dehydrogenase. Moreover, mitochondrial electron transport chain enzymes, succinatedehydrogenase, NADH dehydrogenase, cytochrome c-oxidase, and coenzyme Q-cytochrome reductase activities were also decreased following Cd exposure. PSB administration substantially reduced the mitochondrial membrane potential while inducing significant histological damage. However, PSB treatment significantly reduced Cd-mediated renal damage in rats.

**Conclusion:**

Thus, the present investigation discovered that PSB has ameliorative potential against Cd-induced renal dysfunction in rats.

## Introduction

Cadmium (Cd) is a ubiquitous environmental toxin that is extensively used in industrial and agricultural products (1). Cd is one of the major heavy metals that pollute the ecosystem and has damaging effects on different biological processes in both humans and animals (2). Exposure to Cd can occur through various sources, such as plant-based foods, fertilizers, contaminated water, plastic toys, ceramics, batteries, paints, air, soil, and cigarette smoke (3, 4). The accumulation of Cd in plants mainly occurs through anthropogenic activities such as the application of phosphate fertilizers, wastewater, sewage sludge, and manures. Moreover, the high mobility of soils makes the accumulation of Cd easy in plants. Industries involved in the production of batteries, pigments, coatings, electroplating, and plastic stabilizers are often associated with occupational exposure to Cd.

Moreover, these industries are the sources that make the water contaminated. However, the major Cd occupational exposure occurs through fume inhalation in the cadmium-nickel battery industry. Currently, Cd pollution poses a serious environmental problem. Cd accumulation in the body can affect health, and its exposure may cause hazardous effects such as hepatotoxicity, ototoxicity, carcinogenesis, and nephrotoxicity (5). In living organisms, heavy metals, including Cd, can damage cellular organelles along with components such as lysosomes, cell membranes, endoplasmic reticulum, and mitochondria, as well as some enzymes involved in damage repair, metabolism, and detoxification (6).

The kidneys are the primary target site of Cd exposure in the body. Approximately 50% of the Cd accumulates in the kidney, particularly in the proximal convoluted tubules, which leads to renal dysfunction and chronic kidney disorders (7–10). According to previous research, up to 7% of the world's population suffers from chronic kidney disorders due to Cd exposure (11). Cd induces nephrotoxicity via inflammation, apoptosis, and the generation of ROS in renal tissues (12, 13). Cd exposure generates proinflammatory cytokines such as interleukin IL-1 and TNF-α that trigger inflammation by recruiting innate immune cells (14). These proinflammatory cytokines contribute to inflammation by inducing the expression of adhesion molecules on endothelial cells, causing leukocytes in circulation to adhere to the endothelium. Long-term exposure to Cd significantly decreases the glomerular filtration rate, which can lead to renal failure (15), increased creatinine, blood urea nitrogen (BUN), hydropic swelling, and proximal tubular cells' hypertrophy (12). Mitochondria have long been recognized as a major site for the production of reactive nitrogen species and ROS (16). Furthermore, it has been stated that Cd exposure can cause mitochondrial dysfunction, which may lead to renal failure (17).

Pinostrobin (PSB), or 5-hydroxy-7-methoxy flavanone, is a dietary flavonoid isolated from *Boesenbergia rotunda*. This plant was originally characterized as a medicinal plant due to its potential therapeutical properties such as antiinflammatory (18), antileukemia (19), anticancer (20), antioxidant (21, 22), antimicrobial (23), anti-Alzheimer's (24), and antiviral (25). Cd-induced renal dysfunction occurs due to oxidative stress, inflammation, and apoptosis, as the kidney is the primary target site of Cd-induced toxicity. Therefore, by considering the aforementioned pharmacological activities of PSB, the present investigation was intended to evaluate the potential of PSB to alleviate Cd-induced renal damage in Sprague-Dawley rats.

## Materials and methods

### Chemicals

Cd and PSB were purchased from Sigma-Aldrich (Germany).

### Animals

Mature male Sprague-Dawley rats with an average weight of 185 ± 15 g. The rats were housed at the animal research station of the University of Agriculture, Faisalabad, with a temperature of 25 ± 1°C and a 12-h light/dark cycle maintained throughout the experiments. Furthermore, the rats were given standard feed and water *ad libitum* throughout the whole trial. The rats were acclimatized to the laboratory environment for 7 days before the start of the trial. All animal procedures were conducted in accordance with the approved protocol of the European Union for Animal Care and Experimentation (CEE Council 86/609).

### Experimental layout

A total of 48 male Sprague-Dawley rats were divided into four different groups, 12 in each group, and stored in separate cages. The trial was conducted for 30 days. Group 1 was designated as the control group. Group 2 was given Cd (5 mg/kg) orally. Group 3 was administered Cd (5 mg/kg) and PSB (10 mg/kg) orally until the completion of the experiment (30 days). The rats in group 4 were supplemented with PSB (10 mg/kg). Cd at a dose of 5 mg/kg was given according to a previous investigation [26]. The rats were anesthetized with 60 mg/kg of ketamine and 6 mg/kg of xylazine and dissected after 28 days of treatment. The blood was drawn to analyze the serum profile. Following dissection, the kidneys were removed; one kidney was packaged in a zipper bag and preserved at −80°C for biochemical examination. For histological evaluation, the other kidney was stored in a 10% formalin solution.

### Separation of kidney mitochondria

Mingatto et al.'s approach was used to isolate mitochondria from renal cells (26). The renal tissue was mixed with medium-I (70 mM sucrose, 250 mM mannitol, 50 mM Tris-HCl, 10 mM HEPES, 1 mM EDTA, 120 mM KCl, and pH 7.4). Homogenate was centrifuged for 5 min at 755 × g. The pellets were rinsed in medium-II (250 mM mannitol, 50 mM Tris-HCl, 70 mM sucrose, 10 mM HEPES, and pH 7.4) before being rinsed two times with the same buffer and centrifuged at 13,300 × g for 15 min. Mitochondrial pellets were resuspended in the same medium and utilized for additional investigation.

### Estimation of biochemical markers

CAT activity was evaluated by following the methodology of Aebi (27). The activity of SOD was estimated via the method popularized by Sun et al. (29). Carlberg and Mannervik's (28) technique was used to measure GSR content. GSH-PX activity was estimated by following the method of Lawrence and Burk (29). The MDA level was determined in accordance with the method of Ohkawa et al. (30). ROS level was measured by ELISA kits (Shanghai Enzyme-Linked Biotechnology Co. Ltd., Shanghai, China) as per the manufacturer's instructions.

### Estimation of kidney function markers

Urea, creatinine, and creatinine clearance were determined using standard diagnostic kits (MediScreen kit, France). Urinary KIM-1 and NGAL levels were measured using the KIM-1 Quantikine ELISA Kit and the NGAL Quantikine ELISA Kit, respectively, following the manufacturer's guidelines.

### Estimation of inflammatory indices

By using a tissue lyser device (Tissue Lyser II, Oiagen), renal tissues were homogenized in a cold phosphate buffer (pH 7.4) (31). The commercial kits were used for the measurement of inflammatory indices, i.e., interleukin-6 (IL-6), tumor necrosis factor-α (TNF-α), inducible nitric oxide synthase (iNOS), interleukin-1β (IL-1β), nuclear factor kappa B (NFκB), and cyclooxygenase-2 (COX-2) in renal tissues.

### Estimation of apoptosis markers

RT-qPCR was used to evaluate Bax, Bcl-2, and Caspase-3 expressions. Total RNA isolation was performed using the TRIzol reagent, which was then reverse-transcribed into complementary DNA. Variations in apoptotic markers' expression were observed by 2^−Δ*ΔCT*^ using β-actin as the internal control (32). Sequences (primers) of β-actin and target genes are displayed in Table 1, as reported previously (33).

**Table 1:** Primers sequences of real-time quantitative reverse transcription-polymerase (RT-qPCR).

**Gene**	**Primers 5^′^->3^′^**	**Accession number**
Bax	Forward: GGCCTTTTTGCTACAGGGTT	NM_017059.2
	Reverse: AGCTCCATGTTGTTGTCCAG	
Bcl-2	Forward: ACAACATCGCTCTGTGGAT	NM_016993.1
	Reverse: TCAGAGACAGCCAGGAGAA	
Caspase-3	Forward: ATCCATGGAAGCAAGTCGAT	NM_012922.2
	Reverse: CCTTTTGCTGTGATCTTCCT	
β-actin	Forward: TACAGCTTCACCACCACAGC	NM_031144
	Reverse: GGAACCGCTCATTGCCGATA	

RT-qPCR, reverse transcription-polymerase chain reaction; 3β-HSD, 3β-hydroxysteroid dehydrogenase; 17β-HSD, 17β-hydroxysteroid dehydrogenase; and StAR, steroidogenic acute regulatory protein.

### Assessment of TCA cycle enzymes

Isocitrate dehydrogenase (ICdH) activity was evaluated according to the practice of Bernt and Bergmeyer (34). Succinate dehydrogenase (SDH) activity was determined according to the protocol of Slater and Borner (35). Malate dehydrogenase (MDH) activity was assessed by following the technique of Mehler et al. (36). α-KGDH activity was determined in accordance with the procedure of Reed and Mukherjee (37).

### Analysis of the activity of respiratory chain complexes in the renal mitochondria

Mitochondrial respiratory-chain complex test kits (Suzhou-Comin Biotechnology Ltd., China) were used to evaluate the activity of respiratory chain complexes in renal mitochondria.

### Assessment of mitochondrial membrane potential

Mitochondrial membrane potential (MMP) was determined by measuring the absorption of a cationic fluorescent dye by the mitochondria (38). To incubate the mitochondrial suspension (0.5 mg protein ml-1) with Rh 123 (1.5 M), the tubes were gently shaken for 10 min at 37°C. The Elmer LS-50B luminescence fluorescence spectrophotometer was used to determine fluorescence at emission (490 nm) and excitation (535 nm) wavelengths (38).

### Tissue histology

The renal tissues were fixed in a 10% formalin solution for 48 h. The samples were then desiccated in increasing degrees of alcohol before being embedded in paraffin wax. A rotatory microtome was used to cut 4–5 μm-thick slices, which were then stained with the hematoxylin-eosin stain. In the end, the slides were examined using a light microscope (Nikon, 187842, Japan) at 400X. Microphotography was performed with a Leica LB microscope linked to Olympus Optical Co., Ltd., Japan.

### Statistical analysis

Data were displayed as mean ± SEM. The interpretation of the data was conducted using Minitab software. The data were analyzed using a one-way ANOVA followed by Tukey's test. The significance level was set at a *P*-value of < 0.05.

## Results

### Effect of Cd and PSB on antioxidant enzymes and oxidative markers

Cd exposure considerably (*P* < 0.05) decreased the enzymatic activities of SOD, GSR, and CAT, as well as GSH-PX while markedly (*P* < 0.05) increasing the levels of MDA and ROS compared to the control group. Co-administration of PSB with Cd resulted in a noticeable (*P* < 0.05) increase in the activities of SOD, GSR, and CAT enzymes, as well as GSH-PX while significantly (*P* < 0.05) decreasing MDA and ROS levels compared to the Cd-treated group. The PSB-treated group demonstrated antioxidant enzyme activities, ROS, and MDA levels that were similar to those of the control rats (Table 2).

**Table 2:** Mean ± SEM of biochemical markers in the kidney of control, cadmium, cadmium + pinostrobin, and pinostrobin-supplemented rats.

**Groups**	**CAT (U/g protein)**	**SOD (U/g protein)**	**GSR (Nm NADPH oxidized/min/mg tissues)**	**GSH-PX (U/g protein)**	**ROS**	**MDA (nmol/g tissues)**
Control	8.74 ± 0.22^a^	6.81 ± 0.21^a^	5.86 ± 0.14^a^	23.35 ± 0.68^a^	0.75 ± 0.11^a^	0.44 ± 0.05^a^
Cd	4.24 ± 0.16^c^	2.91 ± 0.13^c^	2.63 ± 0.18^c^	11.42 ± 0.53^c^	7.55 ± 0.19^c^	2.27 ± 0.09^c^
Cd + PSB	6.98 ± 0.12^b^	5.37 ± 0.13^b^	4.92 ± 0.07^b^	18.45 ± 0.61^b^	2.22 ± 0.09^b^	1.26 ± 0.06^b^
PSB	8.65 ± 0.25^a^	6.62 ± 0.22^a^	5.95 ± 0.11^a^	24.18 ± 0.61^a^	0.73 ± 0.08^a^	0.52 ± 0.08^a^

There is a significant difference between means that do not have the same letters.

### Effect of Cd and PSB on kidney function markers

Cd treatment showed remarkable (*P* < 0.05) escalation in urea, KIM-1, and NGAL, as well as creatinine levels, and a noticeable (*P* < 0.05) downregulation in creatinine clearance compared to the control rats. The co-administration of Cd with PSB showed a considerable (*P* < 0.05) reduction in urea, KIM-1, NGAL, and creatinine levels, whereas a substantial increase was noticed in the creatinine clearance when compared to the CD-administrated group. Only the administration of PSB showed levels of renal function markers that were similar to those of the control rats (Table 3).

**Table 3:** Mean ± SEM of renal function markers in control, cadmium-administrated, co-administrated, and pinostrobin-supplemented rats.

**Groups**	**Urea (mg/dl)**	**Creatinine (mg/dl)**	**Creatinine clearance (ml/min)**	**KIM-1 (ng/ml)**	**NGAL (ng/ml)**
Control	15.92 ± 0.45^a^	1.64 ± 0.05^a^	1.56 ± 0.08^a^	0.54 ± 0.07^a^	0.64 ± 0.04^a^
Cd	34.11 ± 0.78^c^	4.47 ± 0.17^c^	0.72 ± 0.32^c^	8.51 ± 0.42^c^	6.14 ± 0.23^c^
Cd + PSB	21.48 ± 0.61^b^	2.06 ± 0.08^b^	1.15 ± 0.06^b^	4.48 ± 0.19^b^	2.21 ± 0.08^b^
PSB	16.48 ± 0.63^a^	1.66 ± 0.06^a^	1.62 ± 0.08^a^	0.65 ± 0.06^a^	0.82 ± 0.06^a^

There is a significant difference between means that do not have the same letters.

### Effect of Cd and PSB on inflammatory cytokines

Cd exposure resulted in a significant (*P* < 0.05) increase in the levels of IL-1β, NF-κB, IL-6, TNF-α, and the activity of iNOS and COX-2 compared to the control group. The co-administration of Cd with PSB led to a noticeable (*P* < 0.05) decline in the levels of these indices compared to the Cd-exposed rats. PSB administration alone demonstrated the same levels of these markers as those observed in the control rats (Table 4).

**Table 4:** Mean ± SEM of inflammatory indices in the renal tissues of control, cadmium-administrated, co-administrated, and pinostrobin-supplemented rats.

**Groups**	**NF-κB (ng/g tissue)**	**TNF-α (ng/g tissue)**	**IL-1β (ng/g tissue)**	**IL-6 (ng/g tissue)**	**iNOS (ng/g tissues)**	**COX-2 (ng/g tissues)**
Control	11.46 ± 0.81^a^	9.28 ± 0.33^a^	35.63 ± 1.24^a^	4.61 ± 0.08^a^	160.07 ± 11.86^a^	27.84 ± 2.78^a^
Cd	49.65 ± 1.53^c^	42.62 ± 1.68^c^	114.41 ± 3.34^c^	19.74 ± 0.49^c^	322.63 ± 12.51^c^	74.01 ± 1.22^c^
Cd+ PSB	26.06 ± 0.91^b^	21.59 ± 0.63^b^	60.37 ± 1.41^b^	9.51 ± 0.64^b^	226.07 ± 9.66^b^	44.12 ± 1.93^b^
PSB	12.29 ± 0.67^a^	9.43 ± 0.67^a^	40.75 ± 2.23^a^	4.95 ± 0.22^a^	169.04 ± 13.51^a^	23.07 ± 1.22^a^

There is a significant difference between means that do not have the same letters.

### Effect of Cd and PSB on apoptotic markers

**Figure 1:**
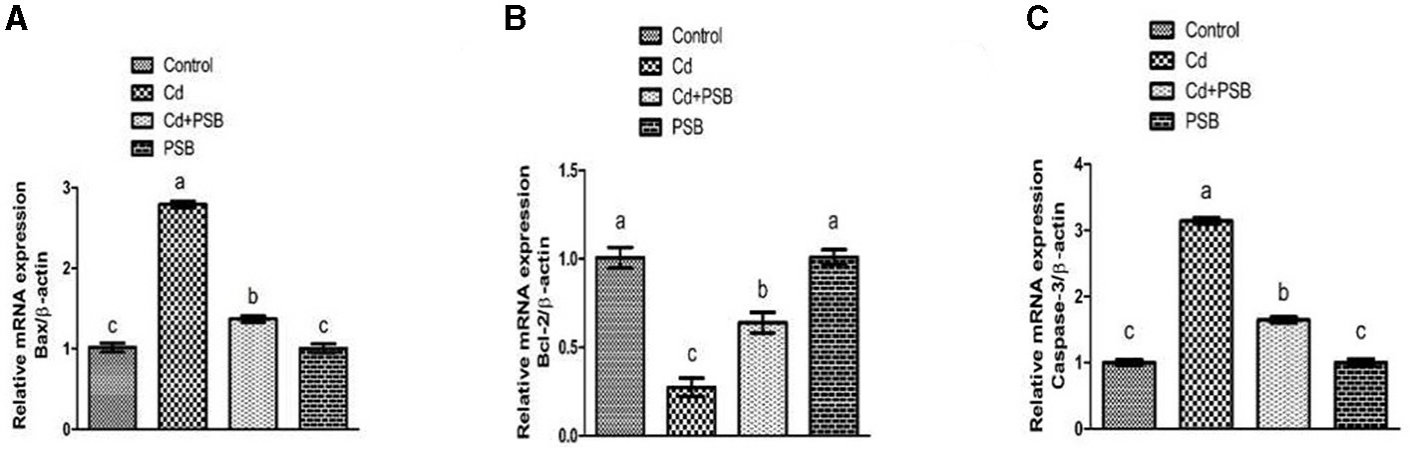
Effect of Cd and PSB on the renal **(A, B)** pro (Bax) and anti-apoptotic (Bcl-2) markers and **(C)** Caspease-3. First bar represents control; Second bar represents Cd treated group; Third bar represents Cd+PSB treated group; Fourth bar represents PSB treated group. Values are expressed as Mean ± SEM (12 rats per group). Values having different superscripts are significantly *p* < 0.05 different from each other.

Cd exposure led to a significant (*P* < 0.05) decrease in the expression of the antiapoptotic marker (Bcl-2) while the expression of the apoptotic marker (caspase-3 & Bax) was upregulated in PSB-administrated rats when compared to the control group. However, co-treatment with Cd and PSB resulted in a significant (*P* < 0.05) reversal of these antiapoptotic and apoptotic marker expressions compared to the Cd-treated group. Administration of PSB alone demonstrated normal expressions of these markers that were comparable to those observed in the control rats (see Figure 1).

### Effect of Cd and PSB on TCA cycle enzymes

Cd intoxication markedly (*P* < 0.05) lowered the TCA cycle enzymes (MDH, ICdH, α-KGDH, and SDH) activities in comparison to the control group. However, co-administration of PSB and Cd led to a significant increase in TCA cycle enzyme activities compared with Cd-administrated rats. The PSB-administrated group showed TCA cycle enzyme activities that were similar to those of the control rats (Table 5).

**Table 5:** Mean ± SEM of tricarboxylic acid cycle enzymes in the renal tissues of control, cadmium-administrated, co-administrated, and pinostrobin-supplemented rats.

**Groups**	**Isocitrate dehydrogenase (units/min/mg of protein)**	**Alpha-ketoglutarate dehydrogenase (units/min/mg of protein)**	**Succinate dehydrogenase (units/min/mg of protein)**	**Malate dehydrogenase (units/min/mg of protein)**
Control	720.30 ± 22.50^a^	177.98 ± 9.73^a^	62.47 ± 6.39^a^	566.63 ± 14.93^a^
Cd	299.51 ± 12.50^c^	56.55 ± 4.80^c^	19.94 ± 3.22^c^	158.61 ± 8.21^c^
Cd+ PSB	623.70 ± 31.00^b^	113.28 ± 10.65^b^	51.76 ± 1.84^b^	383.76 ± 11.72^b^
PSB	728.70 ± 24.70^a^	182.84 ± 13.19^a^	63.52 ± 6.21^a^	573.25 ± 11.11^a^

There is a significant difference between means that do not have the same letters.

### Effect of Cd and PSB on mitochondrial respiratory chain complexes

The co-administration of PSB and Cd substantially restored the activities of mitochondrial respiratory chain complexes when compared to the Cd-treated group. PSB-administrated rats exhibited activities in these complexes that were similar to those of the control rats (Table 6).

**Table 6:** Mean ± SEM of renal mitochondrial respiratory chain complexes along with ΔΨm in control, cadmium-administrated, co-administrated, and pinostrobin-supplemented rats.

**Groups**	**Complex-I (NADH dehydrogenase)**	**Complex-II (succinate-dehydrogenase)**	**Complex-III (coenzyme Q-cytochrome reductase)**	**Complex-IV (cytochrome c oxidase)**	***ΔΨ*m %**
Control	29.72 ± 1.49^a^	68.88 ± 2.27^a^	1.04 ± 0.04^a^	248.07 ± 13.52^a^	82.55 ± 0.62^a^
Cd	8.78 ± 0.55^c^	25.96 ± 2.08^c^	0.26 ± 0.03^c^	130.91 ± 10.45^c^	28.71 ± 1.73^c^
Cd+ PSB	20.93 ± 0.97^b^	36.13 ± 1.97^b^	0.68 ± 0.02^b^	200.11 ± 11.86^b^	64.64 ± 3.78^b^
PSB	28.18 ± 1.41^a^	71.61 ± 3.52^a^	1.08 ± 0.06^a^	237.81 ± 12.38^a^	84.80 ± 4.75^a^

There is a significant difference between means that do not have the same letters.

### Effect of Cd and PSB on mitochondrial membrane potential

The rats exposed to Cd presented a noticeable (*P* < 0.05) depolarization in mitochondrial membrane potential (ΔΨm) when compared to the control rats. However, the co-administration of PSB and Cd partially mitigated the loss of ΔΨm when compared to the Cd-administrated group. Only PSB-administrated rats exhibited mitochondrial membrane potential, which was similar to that observed in the control rats (Table 6).

### Effect of Cd and PSB on histopathology

**Figure 2:**
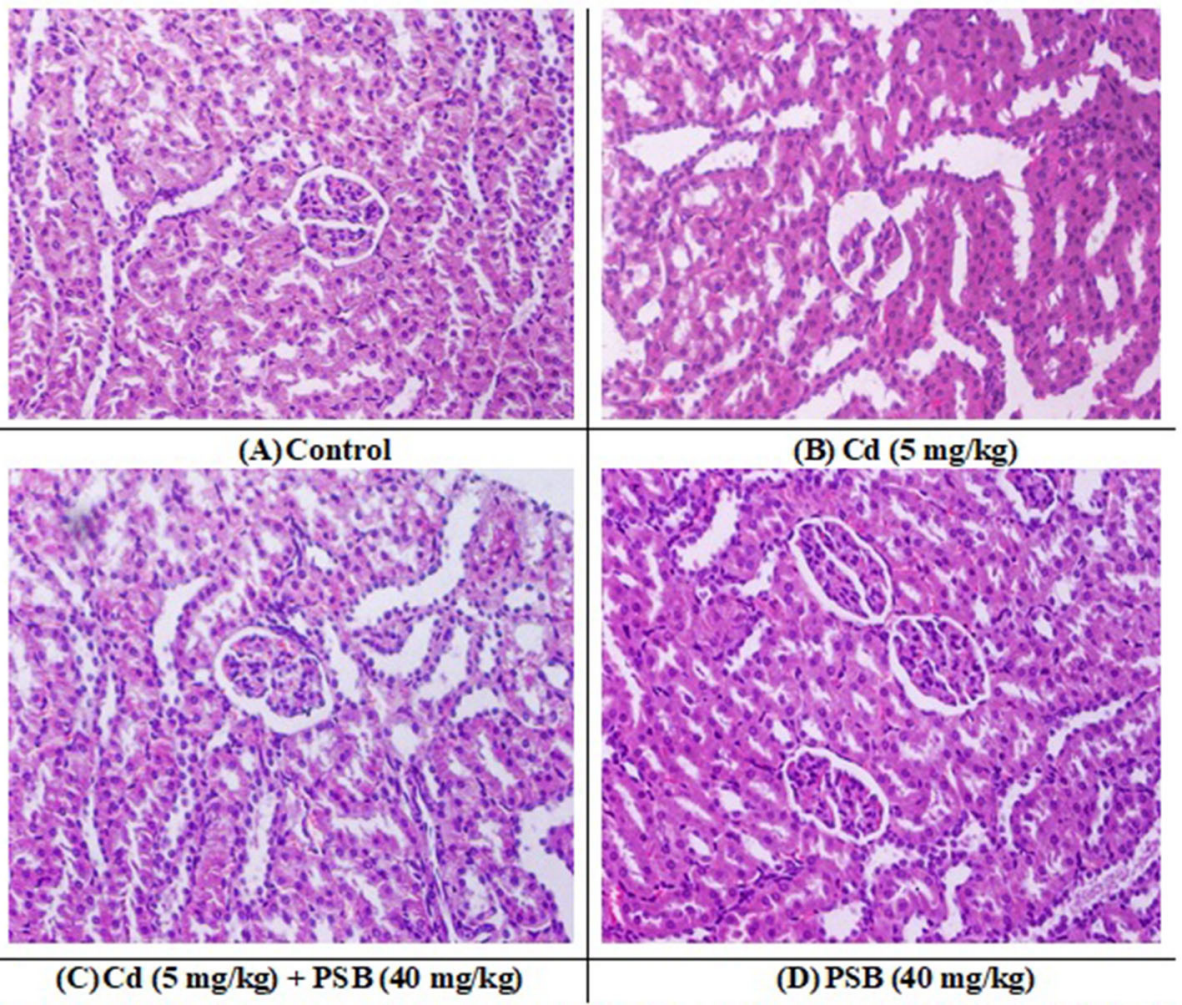
**(A)** control group; displaying normal histological structure (H and E, 400X). **(B)** Cd treated group; showing dilation, vacuolation, degeneration, and widened Bowman's capsule necrosis in the kidney tissues. **(C)** Cd + PSB treated group showing improved histoarchitecture with reduce degenerative architecture in renal epithelium and renal tubules. **(D)** PSB treated group showing normal renal histoarchitecture. Cd, Cadmium; PSB, Pinostrobin.

The kidneys of the rats in the control group showed normal renal tubules and glomeruli. Most of the renal glomeruli appeared regular, without evidence of mesangial cell proliferation or vascular congestion. The shape of the renal tubules ranged from round to oval ducts lined with cuboidal epithelial or polygonal cells. However, kidneys in the Cd-exposed group exhibited a distorted structure, with atrophied glomerular tufts, disruption of Bowman's capsule, and vacuolation in the epithelium of renal tubules. However, the co-treated group displayed mild to moderate vacuolation in the epithelium of the tubules, while the glomeruli were normal in size with a mild level of tuft and distortion in the capillaries. Only administration of PSB showed normal renal tubules and a regular histological profile similar to that observed in the control group (Figure 2 and Table 7).

**Table 7:** Mean ± SEM of renal histology in control, cadmium-administrated, co-administrated, and pinostrobin-supplemented rats.

**Groups**	**Epithelial cell degeneration)**	**Tubular atrophy**	**Glomerular atrophy**	**Leucocytes infiltration**	**Widen Bowman's capsule**
Control	8.77 ± 1.82^a^	4.82 ± 1.93^a^	7.43 ± 1.30^a^	5.95 ± 1.81^a^	6.80 ± 2.01^a^
Cd	76.26 ± 3.77^c^	58.97 ± 3.56^c^	77.34 ± 5.42^c^	64.19 ± 3.60^c^	74.29 ± 1.87^c^
Cd+ PSB	16.17 ± 2.21^b^	13.83 ± 2.68^b^	19.86 ± 1.95^b^	15.58 ± 2.21^b^	13.60 ± 1.75^b^
PSB	8.29 ± 1.37^a^	4.74 ± 1.93^a^	7.41 ± 1.37^a^	5.91 ± 1.80^a^	6.55 ± 2.11^a^

There is a significant difference between means that do not have the same letters.

## Discussion

In underdeveloped nations, Cd is believed to be the most prevalent environmental and accidental contaminant that frequently poses a major threat to both humans and animals (39). Cd is a widely present hazardous industrial pollutant that can accumulate in tissues such as the testis, hepatic tissue, lungs, bones, and renal tissue and cause acute injuries (40). One can become exposed to Cd through various sources, such as plant-based food, fertilizers, contaminated water, ceramics, batteries, and cigarette smoke (3, 4). Cd accumulates in the body and can have various detrimental effects, such as hepatotoxicity, ototoxicity, carcinogenesis, and nephrotoxicity (5). When Cd is released into the cytoplasm, it can induce the production of reactive oxygen species (ROS), lipid peroxidation, depletion of glutathione (GH), cross-linking of proteins, and inflammation. As a result, proinflammatory cytokines accumulate, and kidney cells die, resulting in kidney damage (13, 41). ROS production is the major mechanism of Cd-induced kidney toxicity as it may change cell redox balance (42). However, PSB is well-recognized for its wide variety of pharmaceutical potentials, including analgesic, antioxidant, and antiinflammatory properties (43). PSB has benzo-γ-pyrone in its structure, possibly imparting ROS scavenging potential to PSB and reducing oxidative stress and inflammation (44). Keeping this evidence under consideration, we aimed to assess the therapeutic role of PSB against Cd-induced kidney damage.

Cd exposure led to a significant decrease in the activities of antioxidant enzymes and an increase in the levels of MDA and ROS. This imbalance between ROS and antioxidant enzymes resulted in oxidative stress (45). GPx, CAT, and SOD are critical enzymes involved in the removal of ROS (46). SOD is the primary antioxidant enzyme that converts O^−2^ into hydrogen peroxide (45). Both CAT and GPx then convert hydrogen peroxide into oxygen and H_2_O (47). Previous studies have shown that Cd induces damage in renal cells by producing ROS (48). In earlier investigations, it has been stated that Cd exposure can increase ROS levels, which, as a result, can decrease antioxidant enzyme activities and increase LPO (49). However, the administration of PSB has the potential to reverse these Cd-induced effects on antioxidant enzymes by reducing ROS production. The ability of PSB to protect against oxidative stress may be due to the presence of benzo-γ-pyrone in its structure, which may impart antioxidant potential to PSB (50).

The removal of creatinine and urea from the body is based on the glomerular filtration rate (51). Increased levels of urea can lead to kidney dysfunction, abnormal excretion, and tissue damage (52). A substantial increase in creatinine concentration can be due to the loss of glomerular function and tubular damage in renal tissues (53). KIM-1 and NGAL are promising markers for the assessment of acute renal failure (54). KIM-1 is a type 1 membrane glycoprotein that is highly expressed in proximal tubule cells after exposure to nephrotoxic agents (54). Furthermore, NGAL is a cytoplasmic protein that can be detected in the blood, urine, renal, and proximal-distal tubules following renal damage (55). Therefore, elevated levels of these markers indicate kidney damage. These findings are further supported by the study conducted by Kamel et al. (56), who reported that Cd administration increased the levels of KIM-1, NGAL, creatinine, and urea in rat kidneys. However, PSB treatment normalized these markers and restored KIM-1, NGAL, creatinine, urea, and creatinine clearance. These findings demonstrate the nephroprotective properties of PSB.

NF-κB is a key regulator of the immune response and various inflammatory ailments. It also plays a major role in the activation of inflammatory cytokines such as COX-2, IL-6, TNF-α, IL-1β, and iNOS (57). Previous investigations have proven that heavy metals can directly increase the production of proinflammatory cytokines (58). Moreover, COX-2 and iNOS enzymes modulate the inflammatory response by producing prostaglandin E2 and nitric oxide, respectively (59). Nitric oxide can make the cell more vulnerable to ROS by reducing intracellular glutathione content (60). Several previous studies have indicated that Cd exposure in kidney tissues can activate NF- κB, which increases IL-1, IL-6, and TNF-α while decreasing IL-10 (61–63). Conversely, PSB treatment downregulated NF-κB expression and significantly reduced the levels of IL-6, TNF-α, and IL-1β. PSB significantly suppressed iNOS and COX-2 activities, which might be attributed to its ring structure, as confirmed by the earlier study that reported the antiinflammatory potential of flavonoids to be attributed to the non-methoxylation of the 3′-hydroxyl groups on the B-ring or methoxylation of the 5- or 7-hydroxyl groups on the A-ring (64).

Apoptosis occurs due to an imbalance in apoptotic and antiapoptotic proteins through mitochondrial-independent and dependent pathways (65). Downregulation of Bcl-2 and upregulation of Bax severely change the stability of the mitochondrial membrane (66). Bax and Bcl-2 facilitate the discharge of cytochrome c from mitochondria, which initiates the basic apoptotic pathway (67, 68). Caspase-3 is identified as a crucial apoptosis mediator, as it starts the apoptotic mechanism by stimulating other caspase enzymes (69). A previous investigation supports our results that Cd exposure significantly increased the expression of Caspase-3 and Bax while decreasing the level of Bcl-2 in the renal tissues of rats (70, 71). Cd treatment led to a substantial increase in Bax and Caspase-3 expression while reducing the levels of Bcl-2. However, PSB treatment prevented Cd-induced renal apoptosis by suppressing the activation of Caspase-3 and downregulating Bax, leading to an upregulation of Bcl-2. The outcomes of our investigation indicate that PSB possesses antiapoptotic potential against Cd exposure.

Mitochondria are known as the powerhouse of the cell due to their ability to produce ATP through the process of oxidative phosphorylation (OP) (72). Mitochondria are the most critical organelles and play a central role in maintaining cellular homeostasis. Therefore, mitochondrial dysfunction may lead to tissue or cellular damage (73). Through the TCA cycle, mitochondrial enzymes catalyze the oxidation of various substrates, reducing their equivalents. These electrons are transferred across the respiratory chain to generate ATP through OP (74, 75). The study conducted by Ijaz et al. (17) demonstrated that Cd exposure decreased TCA cycle enzyme activities, which eventually caused mitochondrial impairment in the kidneys of rats. Conversely, co-administration of PSB with Cd reversed TCA cycle enzyme activities, possibly by downregulating OS.

In mitochondria, the electron transport chain (ETC) is responsible for oxidative phosphorylation, which uses fatty acids and pyruvate to produce ATP. Under physiological conditions, ETC is supposed to be the primary cause of ROS generation (76). According to earlier studies, the accumulation of intracellular ROS is a major cause of mitochondrial ETC damage (77, 78). Mitochondrial ETC can be damaged, which is usually indicated by OS and reduced ATP generation (79). When the mitochondrial electron transport chain's transmission is interrupted, ATP production is affected, which results in mitochondrial damage (80). In an earlier study, Belyaeva et al. (81) stated that both membrane permeability and mitochondrial ETC are the major targets of Cd-induced mitochondrial damage. However, PSB treatment probably diminished mitochondrial dysfunction by increasing complex (I-V) activities to their normal ranges due to its mitigative effects.

Cd exposure resulted in mitochondrial membrane potential collapse (ΔΨm). ΔΨm produced via mitochondrial complexes during oxidative phosphorylation is important in energy storage. ΔΨm also plays a critical role in maintaining mitochondrial homeostasis (82). Overproduction of ROS can interrupt multiple mitochondrial processes, i.e., mitochondrial swelling, MMP collapse, and reduced dehydrogenase activity (83).

The maintenance of ΔΨm is also essential for the movement of mitochondria (84). According to a previous study, the anterograde mobility of mitochondria is crucial for their proper functioning (85). According to previous research, the downregulation of ETC reduces proton efflux through the inner membrane of mitochondria, which disrupts the depolarization of ΔΨm (86). Our findings suggested that PSB has the potential to restore Cd-induced ΔΨm loss by upregulating the activities of ETC complexes. Moreover, PSB administration significantly restored Cd-mediated ΔΨm. This normalization is possibly due to an increase in the activity of the ETC complex.

Cd exposure resulted in degeneration of tubular cells and epithelial cells, glomerular atrophy, leucocyte infiltration, and disruption of Bowman's capsule in the proximal renal tubules. These histomorphological changes could reflect direct impairment in renal tissues. Our findings are further supported by the investigation of Kamel et al. (56), who reported that Cd administration led to histomorphological changes in renal tissues, such as tubular and glomerular atrophy. It is speculated that histomorphological changes may be due to the excessive production of ROS caused by Cd exposure (87), which resulted in oxidative impairment (88) and morphological changes in renal tissue. However, the PSB-co-treated group displayed mild to moderate vacuolation in the epithelium of tubules. Furthermore, Cd co-administration with PSB significantly mitigated the above-mentioned histopathological damage. This may be attributed to the antioxidant capability of PSB, which significantly decreased oxidative stress, leading to a decrease in pathological alterations.

## Conclusion

In conclusion, our research demonstrated that PSB supplementation potentially attenuated the Cd-induced hazardous effects on respiratory chain complexes, urea, creatinine, antioxidant enzymes, creatinine clearance, TCA cycle enzymes, and ΔΨm. Furthermore, PSB administration considerably reduced Cd-induced renal dysfunction by mitigating renal oxidative stress, apoptosis, and inflammatory reactions. PSB regulates renal functions by restoring TCA cycle enzyme activities and ETC complexes. These nephroprotective effects of PSB against Cd-mediated nephrotoxicity may be attributed to its antiinflammatory, antiapoptotic, and antioxidant nature. Taken together, it can be concluded that PSB may have some clinical applications in the future to cure Cd-induced renal dysfunctions in humans.

### Limitation

The limitation of the study is that it was conducted on animal models, and it is necessary to conduct clinical trials in the future to determine the effectiveness and safety of PSB in humans.

## Data availability statement

The original contributions presented in the study are included in the article/supplementary material, further inquiries can be directed to the corresponding author.

## Ethics statement

This animal study was authorized by the Institutional Biosafety/Bioethics Committee (IBC) of the University of Agriculture, Faisalabad, according to the (CEE Council 86/609) protocol.

## Author contributions

MI, TA, SR, and SS perceived the idea and planned the investigation. AH and RA conducted the experiments. HA assisted in the statistical analysis. MI, AN, MA-O, SR, TA, MB, HS, SS, and AH wrote the manuscript. All authors have read and authorized the final version of the manuscript.
